# Health care providers’ early experiences of assisted dying in Aotearoa New Zealand: an evolving clinical service

**DOI:** 10.1186/s12904-023-01222-4

**Published:** 2023-07-22

**Authors:** Jeanne Snelling, Jessica Young, Sophie Beaumont, Kate Diesfeld, Ben White, Lindy Willmott, Jacqualine Robinson, Tess Moeke-Maxwell

**Affiliations:** 1 University of Otago, Dunedin, New Zealand; 2 Te Herenga Waka – Victoria University of Wellington, Wellington, New Zealand; 3 Auckland University of Technology, Auckland, New Zealand; 4 Queensland University of Technology, Brisbane, Australia; 5 University of Auckland, Auckland, New Zealand

**Keywords:** Assisted dying, Euthanasia, End of life policy and practice, Provider perspectives

## Abstract

**Background:**

In November 2021, assisted dying (AD) became lawful in Aotearoa New Zealand. A terminally ill person may now request, and receive, pharmacological assistance (self-administered or provided by a medical practitioner/nurse practitioner) to end their life, subject to specific legal criteria and processes. Exploring the experiences of health providers in the initial stage of the implementation of the End of Life Choice Act 2019 is vital to inform the ongoing development of safe and effective AD practice, policy and law.

**Aim:**

To explore the early experiences of health care providers (HCPs) who do and do not provide AD services seven months after legalisation of AD to provide the first empirical account of how the AD service is operating in New Zealand’s distinctive healthcare environment and cultural context.

**Design:**

Qualitative exploratory design using semi-structured individual and focus group interviewing with a range of HCPs.

**Results:**

Twenty-six HCPs participated in the study. Through a process of thematic analysis four key themes were identified: (1) Difference in organisational response to AD; (2) challenges in applying the law; (3) experiences at the coal face; and (4) functionality of the AD system.

**Conclusion:**

A range of barriers and enablers to successful implementation of AD were described. Adoption of open and transparent organisational policies, ongoing education of the workforce, and measures to reduce stigma associated with AD are necessary to facilitate high quality AD service provision. Future research into the factors that influence responses to, and experience of AD; the impact of institutional objection; and the extent to which HCP perspectives evolve over time would be beneficial. In addition, further research into the integration of AD within Māori health organisations is required.

**Supplementary Information:**

The online version contains supplementary material available at 10.1186/s12904-023-01222-4.

## Introduction

In November 2021, Aotearoa New Zealand’s End of Life Choice Act 2019 (the Act) came into force, making it lawful for a competent terminally ill person to request and receive pharmacological assistance to end their life, subject to specific legal criteria and processes (see Table 1). The Act defines ‘assisted dying’ (AD) (sometimes also called euthanasia, medical aid-in-dying or physician-assisted dying) as including administration by a medical practitioner or nurse practitioner of medication to hasten a person’s death, or alternatively, the self-administration of medication to hasten a person’s death.

**Table 1:**
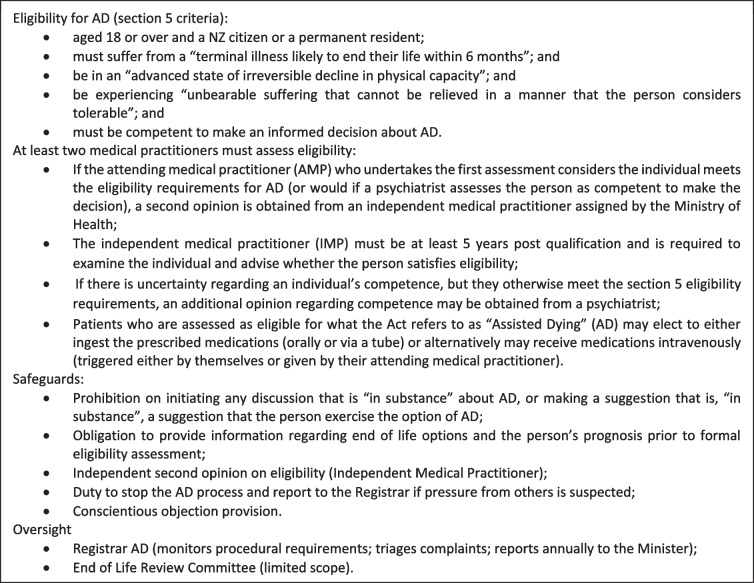
End of life choice act 2019 [1]

Prior to the introduction of the Act, legalisation of AD was a contested issue in New Zealand. Several earlier attempts to legalise AD (both law reform and a High Court challenge to the prohibition on AD) were all unsuccessful [2]. The current Act, the result of a private members’ bill drawn from the ballot box in 2017, was subsequently made contingent on a public referendum. The Act came into effect in November 2021, 12 months after 65.1% of referendum voters supported its introduction. The Ministry of Health (MOH) is required to review the Act’s operation within three years of it coming into force [2].

The rapid transition from AD being merely proposed in law to its integration into clinical practice, as well as the complexities associated with introducing a new and ethically charged clinical service nationwide, motivated this study. Translating legislation into practice can be challenging, with research suggesting that the implementation of AD services in particular can have a significant impact on clinical practice [3].

New Zealand’s Act was primarily influenced by Canadian law as well as that of the Australian State of Victoria, which introduced legislation in 2016 and 2017 respectively [2]. Multiple studies from these two jurisdictions highlight the importance of research into clinicians’ and patients’ experiences when AD services are first introduced to inform the development of law, policy and practice [4–6].

In Canada, studies on early experiences revealed both predicted and unpredicted issues [7]. Evidence shows that early AD providers are challenged by: a lack of clear guidelines and protocols; role ambiguity; evaluating capacity and consent; conscientious objection; and a lack of interprofessional collaboration [4, 8, 9]. Another study involving the early experiences of Canadian physicians found some experienced an unexpected emotional toll from the AD service rollout, and in some instances felt burdened by time pressures [7]. A recent meta-synthesis of studies exploring nurses’ experiences with AD reports that nurses, who are generally at the forefront of patient care including AD, need clear policy, support, communication training and emotional protection [10].

In Victoria, the first Australian state to legalise voluntary assisted dying, an 18-month implementation period was established to ensure all key stakeholders were adequately prepared for the new service [11]. This staged roll-out was government-led, involved extensive consultation, and has been described as facilitating the successful integration of AD within the Victorian health system [12]. Despite these initiatives, research has identified key challenges in the early stages of implementation in Victoria, specifically: the need to balance tensions between policy goals and legislative safeguards; the translation of law into practice; and the ongoing management of conscientious objection [3]. Difficulties during the first year of AD being available in Victoria were highlighted in a recent study exploring the perspectives of doctors involved with AD [13]. They found that the statutory requirement for all AD consults to be conducted in-person created additional burdens for health professionals and terminally ill patients seeking AD. In addition, doctors described how the statutory prohibition on physicians raising the topic of AD with patients (the so-called ‘gag’ clause) clashed with their perceptions of good medical practice, preventing them from providing patients with information regarding the range of available treatment options. Finally, physicians reported systems issues including difficulties with the AD system portal that created difficulties in assisting patients to access AD.

The focus of this study is to explore the early experiences of health care providers (HCPs) who do, and do not, provide AD services. This latter category of “non-providers” is a diverse group including HCPs who are not opposed AD but who, for various reasons, do not provide or participate in AD service provision, as well as HCPs who do not provide or participate in AD because they are philosophically opposed to AD. This study provides the first empirical account of how the AD service has been implemented and is operating in New Zealand’s distinctive healthcare environment and cultural context. New Zealand’s health system is characterised by being primarily publicly funded, although hospice services and aged residential care (ARC) residences receive only partial funding. In addition, New Zealand is increasingly committed to biculturalism and honouring obligations owed to its indigenous Māori peoples under Te Tiriti o Waitangi (the Treaty of Waitangi) which requires the provision of culturally sensitive and equitable health care services. Currently it is not known how New Zealand’s new AD law is being integrated into clinical practice. This study aims to explore the experiences of a range of HCPs in the context of New Zealand’s particular healthcare system and cultural environment. However, it should be noted that this early study focused on mainstream health services and did not expect to capture information about delivering equitable AD services to New Zealand’s indigenous population from a Māori organisational perspective.

## Methods

An exploratory qualitative study design was adopted. Participants were recruited through a survey conducted to identify research priorities regarding the implementation and delivery of AD in New Zealand [14]. At the end of the survey participants were invited to participate in a one-on-one interview to explore their experiences of AD. A process of purposeful sampling was undertaken to ensure a representation of various disciplines and clinical settings (see Tables 2 and 4). Participants were offered individual interviews via zoom. In addition, one survey participant offered to organise a focus group with colleagues from their provincial health organisation. This focus group of ten people was conducted via zoom, with all participants gathered onsite. All of the interviews and focus group were conducted by the second author, an experienced qualitative researcher, using an interview guide to ensure consistent processes throughout. To maximise individual contributions, participants were given an opportunity to provide any further comments after the interview or focus group to the interviewer via email. The interviews were professionally transcribed, and participants were given an opportunity to review their transcripts and to make any changes or clarifications they might wish to make.

**Table 2:** Participant numbers & job titles

Participant Number	Job Title
1	Nurse, Hospice
2	Nurse Practitioner (NP), ARC
3	Medical Director, Hospice (1)
4	Specialist, Retired
5	Implementation Lead, Hospital
6	Nursing Manager, ARC
7	Specialist, Hospital (1)
8	Specialist, Hospital (2)
9	General Practitioner (GP), ARC
10	Clinical Nurse Specialist, Hospice
11	NP, Primary Care
12	Specialist, Hospital (3)
13	GP & Practice Owner
14	Medical Director & Practice Owner, GP
15	Medical Director, Hospice (2)
16	GP

Data was collected between 28 June and 28 July 2022. Interviews were on average one hour in duration (range: 30–75 min). Participants included both providers who do and do not administer AD. The interviewer obtained demographic information (see Table 3) before exploring participants’ experiences of the implementation of AD and the impact this has had on their clinical practice (see Table 3).

**Table 3:**
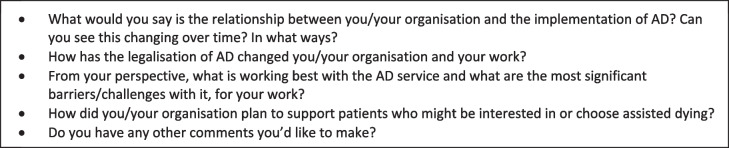
Interview questions

### Data analysis

The transcripts were analysed using inductive thematic analysis, an approach that enables ideas to be identified from the data. Reflexive thematic analysis posits that researcher subjectivity is the primary resource for analysis because knowledge generation is always inherently subjective, co-constructed and situated [15]. We incorporated several steps to ensure the rigour and reflexivity of our approach. Using open coding, two authors separately reviewed all transcripts to identify concepts. Following the process outlined by Braun and Clarke [16], initial codes were inductively and independently produced (second author using NVivo software and first author using manual open coding) with each researcher identifying codes and categories. To enhance rigour, a third researcher read all of the transcripts, whilst a fourth read and coded a sample of transcripts, and the emerging themes were considered as a group. After this preliminary analysis and discussion, the first three authors again discussed the codes, the potential influence of their subjective views on AD, and the possible themes, which were grouped and regrouped until there was agreement. An audit trail of audio recordings, transcripts, codes and the comprising data, memos and thematic developments were kept. The final thematics were checked with the multi-disciplinary research team for their credibility and explanatory power.

### Ethical considerations

All participants were provided with information regarding the aim of the study and informed consent obtained. Because the interviewer was known to some of the interviewees, care was taken to ensure the interview guide was followed. To protect anonymity participants are referred to by their job titles only, and the organisations have not been disclosed (see Table 2). The study was approved by Te Herenga Waka—Victoria University of Wellington (NZ) Ethics Committee (#30,250).

## Results

Participants represented a range of clinical practice areas, but primarily medicine and nursing (*n* = 26), with slightly more females than males represented (see Tables 2 and 4). The single focus group of ten was drawn from one organisation and comprised a Clinical Nursing Director, two health care assistants, a medical student, a care coordinator, with the remaining participants Registered Nurses.

**Table 4:** Demographic characteristics of participants

Category	Subcategory	Frequency (N)	Percent (%)
Gender			
	Woman/Wāhine	19	73.1
	Man/Tāne	7	26.9
Ethnicity			
	New Zealand European/Pākehā	16	61.5
	Māori	1	3.8
	Other European	7	26.9
	Samoan	1	3.8
	Chinese	3	11.5
	Other	1	3.8
*Participants could provide multiple self-identified ethnicities*			
Age			
	25–34	2	7.7
	35–44	4	15.4
	45–54	9	34.6
	55–64	7	26.9
	65–74	3	11.5
	75 +	1	3.8
Role			
	Physicians		
	*GP*	6	23.1
	*Palliative Care Physician*	4	15.4
	*Medical Director*	3	11.5
	*Consultant Cardiologist*	1	3.8
	*Medical Oncologist*	1	3.8
	Nursing		
	*Registered Nurse*	5	19.2
	*Community Hospice Nurse*	3	11.5
	*Nurse Practitioner*	2	7.7
	*Clinical Nurse Specialist*	1	3.8
	*Clinical Nursing Director*	1	3.8
	Other		
	*Healthcare Assistants*	2	7.7
	*Practice Owner*	2	7.7
	*Patient Experience Manager*	1	3.8
	*Hospital Operation Service Manager*	1	3.8
	*Medical student*	1	3.8
*Note: Participants could hold multiple roles*			

All % rounded to 1dp

While not directly asked, participants generally indicated their individual views regarding legalisation of AD during their interview, which reflected significant diversity. After analysing the data it became apparent that participants fell into one of several groups. First, those who were, and remained, opposed to legalisation and who did not participate in AD on the grounds of conscience (conscientious objectors). Another subset of participants originally opposed legalisation, but nevertheless accepted that because it was now lawful, it needed to be accommodated in clinical practice. HCPs in this latter group facilitated aspects of care for people requesting or receiving AD, but were not directly involved with the AD procedure (reluctant AD facilitators). Conversely, there were participants who were supportive of legalisation, and either directly involved in AD service provision (AD participators), or alternatively supportive but not directly involved for various reasons, e.g. because AD was not permitted by their organisation, or the legislation did not allow them to provide AD (non-participator allies).

Four main themes were identified contributing to the overall theme of an evolving service: (1) difference in organisational response to AD; (2) challenges in applying the law; (3) experiences of AD at the “coal face”; and (4) functionality of the AD system.

### Difference in organisational responses to AD

When the AD service was first implemented in November 2021, the health system was under additional pressure as a result of the COVID-19 pandemic. Some participants discussed the implications of this, including challenges associated with high patient admissions, staff shortages, constrained budgets as well as the variation in the provision of, and access to, palliative and hospice services. In practice, this meant any additional service was difficult to establish and integrate at that time. Overall, participants reported significant variation in how AD has been, and continues to be, implemented across different settings, i.e. hospitals, hospices, general practices, and ARC facilities.

#### Variation in organisational response and logistics

Participants described a range of organisational responses, from being “almost obstructive” (GP & Practice Owner); and “burying their heads in the sand” (GP); to making it easy for staff to abide by the law (Medical Director & Practice Owner, GP) and “really supportive practice” (GP). One participant exposed to this spectrum of responses reported:

… there’s a range of views. Some of the companies have said, yep, we’re on board with this, this is the service, our staff aren’t going to be the practitioners but we’re happy for this to happen, this is the person’s home, it should happen here. Others have said we’re supportive of this but it’s not happening here. And, others have said we don’t want to be part of this at all. (NP, ARC)

Participants described how some organisations had proactively considered how AD would impact their organisation and implemented strategies in advance of the law coming into effect:

The Chief Medical Officer was very invested in this so, the mandate was high. There was a short time frame and so we took a community approach. We involved general practice, we involved the hospital ... And aged care, tried to involve aged care as well. So we just had a working group. We had the resources and we met every other week to, over a period of time, just to figure it all out (Implementation Lead, Hospital).

Some organisations mandated staff training on the Act. Participants gave examples of proactive approaches which they perceived created an open and safe learning environment for staff. While some staff were not involved in AD due to their organisation’s position, others chose not to be involved for personal reasons, such as religious convictions. One ARC manager also described how they actively supported staff who were uncomfortable with AD:

They were involved up to the point where they felt they could no longer deliver safe care. And, for me it was around checking in with them daily and just having that really open conversation. And, hearing from them, you know, and giving them the opportunity to, sort of, say, you know what I’m actually okay with this now. Or, no, I’m not okay I need to step away ... And, they decided that even though it was against their cultural and also religious beliefs, that they found that it was actually a privilege to have been involved (Nursing Manager, ARC).

Not all organisations were pro-active in supporting AD, with one participant commenting on the variations across services and different rates of organisational adoption of, and comfort with, AD:

I think the interplay between organisational alignments, perspectives, culture and individual preferences for assisted dying is in a turbulent state … there’s different rates of adoption and different rates of organisational comfort and understanding of how it fits into their paradigm and their value set. And if that’s not signalled clearly and is not simply a what someone in a position of power’s perspective is, as opposed to what the philosophy and ethos of the organisation is, you’ve got a real problem. (Specialist, Hospital (3))

Some participants’ accounts, including those that were comparatively well prepared in advance of the Act’s introduction, reflected the challenge of accommodating a request for AD. One participant described the difficulty of identifying an appropriate location for AD when a patient did not want, or was unable, to die in their own home:

Like where would we do something like this? So there was a lot of like ‘oh not here thanks, no not here’. (Implementation Lead, Hospital).

This same participant observed how this experience led to organisational change to improve end of life care in general, signalling a positive evolution in clinical care. This included recognition that there needed to be a suitable place where AD could occur, either at a regional or main hospital if necessary. The benefits of AD occurring in an environment where staff were used to caring for patients with palliative care needs as well as providing consistency in delivery of AD was acknowledged:

Better that it be consistently one place so that if it were to ever happen again that it would happen there. And so in terms of ripples well they’ve, they’ve always wanted to have a palliative care space. We don’t have one. So now this has been written into the business case for a palliative care space, with also for assisted dying. ‘Cause there’s real recognition that, you know, this is, yeah ideally you’d have a private space, you know? Where people can have space next door. You can welcome the family. You know, it should be inviting. That was always, we always wanted something, somewhere, something inviting (Implementation Lead, Hospital).

The variation in organisational responses to AD was often attributed to the type and size of the organisation, as well as the composition and values of the leadership team. A difficulty with such variation in AD adoption meant that AD providers (and arguably patients) did not know what to expect each time a new site was encountered. The variation was further complicated by the relative speed of the rollout as some organisation’s response to AD was “cobbled together” (Specialist, Hospital (1)).

#### Institutional objection

Although the Act establishes a person’s right to request AD in New Zealand, it does not explicitly require organisations to provide AD services. While conscientious objection is generally an individual matter, some institutions in New Zealand (both privately and publicly funded) have opted not to provide AD services, adopting an ‘institutional objection’ (IO), regardless of whether or not individual HCPs are willing to provide AD services (15). Some participants, including those who worked in hospice, thought that the hospices’ position may shift over time in response to public demand. Some participants reflected a commitment to caring for patients seeking AD, even if the practice of AD was not supported within their organisation:

We’ve been really clear that we support the patients regardless of their decision, and wherever they are in that process ... well, we’re going to continue to work on your pain until that day. We’re going to continue to support you (Specialist, Hospital (1)).

Similarly, another participant described how, although their organisation will not provide AD, it is willing to continue to provide pre and post death care for patients and family/whānau:

So there’s really that tension between they don’t want staff in the house or in the venue just prior to administration and at the time of administration. But quite happy to support either side. (Clinical Nurse Specialist, Hospice)

Some participants expressed the view that facilities that receive even partial state funding should not be allowed to object to people dying by AD on site:

I do feel we’re missing a trick with rest homes because I think so many die in there now. And if the hospitals can allow this, why not the rest homes? There are subsidies and all sorts of things involved in that too. Just like hospices. (Specialist, Retired)

In addition, some expressed concern that organisations that object to AD risk negatively impacting patients who seek AD by virtue of the implicit message that:

[It’s] your right to do this, but actually you can’t die on our premises. What that does to the status and humanity of that person and actually, it “others” them… The organisation says we believe that people should have a good death, but only as we define what a good death is. So they’re actually making a judgement on this person that this is not a good death because if it was a good death we would support it, you know? And I think that’s terrible and I’ve spoken to several people in my practice who have felt like that, who have picked up that sense of almost shame that they are asking for this. (Specialist, Hospital (3))

Some participants reported how some patients and their families described feelings of guilt, or shame, or felt “brushed off” when seeking AD in institutions that objected to AD. One participant suggested that organisations that directly prevent or discourage staff from discussing AD with patients miss opportunities to explore, and alleviate, a patients’ distress:

… they are probably not able to do their job as well as they would like to… They can’t allay some of the fears that would be behind the requests as well. So I think they’re actually disarming themselves from some really good clinical stuff that would help the patient on their journey no matter if they go through assisted death or not. (Medical Director, Hospice (2))

One participant noted the irony that while hospices in New Zealand (with one exception) do not support AD service, because of their expertise they are uniquely well-placed to facilitate AD conversations.

I always thought that we in palliative care would be best placed under the present legislation to be the ones counselling patients seeking assisted dying. Even if we weren’t doing it we could be the sheep gate because we’re better at, well some are, better at the prognostication, communication etc. (Medical Director, Hospice (1)).

While some AD providers reported difficulties communicating with or accessing institutions that objected to AD, others had found them cooperative.

#### Conscientious objection

Participants generally reported a high level of awareness of the duty of medical practitioners with a conscientious objection to AD to refer patients who request AD to the SCENZ Group (Support and Consultation for End of Life in New Zealand) to obtain a replacement medical practitioner. However, one participant had witnessed a lack of knowledge in this respect:

I think a lot of people when it’s brought up, you know, the clinicians panic and then they’re, like, oh my goodness what do I do now? So, one was, like, I better call Medical Protection to get some advice about what I need to do here. And, it’s, like, no, no, no, you don’t as long as you just do what the law says… I think once you talk to people and tell them what their obligations are, it’s fine. (Specialist, Hospital (1))

Interestingly, although some HCPs were philosophically opposed to AD, they nevertheless co-operated with AD providers, such as by supplying clinical information when requested. While such services qualify for specific funding, some HCPs reconciled their personal opposition to AD by refusing payment for their services:

In my experience, there have been GPs who’ve said, look, I really don’t like doing this, I’m really against this. But they will provide you with a letter outlining the prognosis and stuff which we have to get, obviously. They just won’t, don’t want to be paid for it. (GP, ARC)

### Challenges in applying the law

The right to request and receive AD in New Zealand is subject to a range of statutory eligibility requirements and safeguards to ensure only eligible patients receive access to AD (see Table 1). Many participants discussed aspects of the law, particularly the safeguards and assessing eligibility.

#### Prohibition on raising assisted dying

One safeguard discussed by some participants was the prohibition on HCPs initiating any discussion that is “in substance” about AD, or making a suggestion that is “in substance” a suggestion that the person exercise the option of AD. While one participant found the prohibition reassuring in that they could be confident that the decision to seek AD was completely patient-driven, others thought that it is unethical not to be able to discuss AD with patients. Against the background of poor understanding in general of end of life care options particularly for some groups, one participant referred to the prohibition as “muzzling, stifling, censorship of clinicians” (Specialist, Hospital (3)). This participant also spoke about what they considered was an inherent contradiction within the Act:

This is what this law is saying. Saying that clinicians are not to be trusted. And yet we are tasked with safeguarding coercion by screening for it in families. So we are trusted to be able to have this kind of ability to pick up coercion in families, but we are not trusted that we are not coercive ourselves. (Specialist, Hospital (3))

Another participant noted a lack of understanding regarding the scope of the prohibition amongst staff in general:

Some of our staff feel like, that a person should be raising the topic of assisted dying every time they have an interaction before our staff can legally have a conversation. I’m like, no! That’s not the case.(Clinical Nurse Specialist, Hospice)

#### Eligibility: prognosis and likelihood of death within six months

Some AD providers reported different challenges depending on where the patient is on the dying trajectory, as well as the nature of the disease they are suffering from. For example, one AD provider participant observed that as more time has passed since the law came into effect they are dealing with a different type of patient. In the early days/months of AD becoming legal “people were rushing to it because they didn’t have much time left”. (Specialist, Hospital (2)) For some of these people, the extent of their illness and subsequent deterioration meant they were unable to retain the degree of competence required to consent to AD administration. That participant also reported now seeing patients with terminal illness wanting to plan ahead and to get their “ducks lined up”, but who are not likely to die within 6 months, or whose symptoms are relatively controlled and are not yet experiencing “unbearable suffering”.

Another issue identified by some participants was the difference between making a prognosis for *a* particular patient, versus applying an average prognosis for a particular disease in a specific patient population. For example, one participant alluded to the tension between a narrow focus on disease progression according to population statistics, versus a more holistic approach based on an individual patient:

I’ve had an issue with siloing in medicine and super specialisation. And I just think this may come back and bite us with people who are dying because they’ll get specialists who are reviewing for one tiny aspect of them not knowing really it’s the whole person who’s dying. (Specialist, Retired)

Another issue reported by participants is the difficulty associated with prognostication for particular chronic conditions, such as heart failure or respiratory failure. These illnesses are often characterised by potentially life-threatening exacerbations but individuals may, after medical intervention, experience relatively “stable” periods although still experiencing ongoing and significant impairment. For this group suffering chronic illness, the nature of “unbearable suffering” experienced is partly existential in that they have “had enough” of living with the limitations of their condition.

#### Eligibility: “unbearable suffering”

The Act defines unbearable suffering as when the person “experiences unbearable suffering that cannot be relieved in a manner that the person considers tolerable”. Many participants described how they adopted a subjective approach to the legal test of unbearable suffering:

I think it’s really important that doctors remove themselves a little bit on that and actually make sure patients understand the definition of what unbearable suffering is and allow them to make that decision … his definition was more kind of a spiritual thing. In that he’s, you know, he loves the outdoors … He found it really upsetting to have to be inside looking out and not be able to go out. That was his, that was his quality of life. So we, that’s what we defined as being unbearable suffering. Because at the time he didn’t have a lot of the other things that you’d traditionally associate with unbearable suffering. (GP & Practice Owner)

Another participant considered the eligibility criteria a strength of the legislation, but reported experiencing challenges in assessing “unbearable suffering”:

[Unbearable suffering is] an area of controversy and confusion. And I think, so that’s one of the areas where as an AMP [attending medical practitioner], I’ve had the IMP [independent medical practitioner] disagree with me … having a second opinion I think works quite well. (Medical Director & Practice Owner, GP)

#### Statutory requirement for an eligible person to set a date for AD

Unlike other countries, after a person is assessed as eligible for AD in New Zealand they must nominate a specific date and time to undergo AD, although they may subsequently decide not to receive the medication, or may defer the procedure to a later date (provided it is not more than six months after the initial date). Several participants discussed the impact on patients of requiring patients to set a date for undergoing an assisted death. They described how many patients set it as far out as possible, knowing they could bring it forward, but not always choosing to do so, or losing competence before that date. According to some providers, knowing that they could initiate the procedure served as reassurance for the patient, but did not mean a person would necessarily undergo the procedure. One participant wondered if a better option would be for patients *not* to be required to set a date if doing so created stress:

I think a better option would be not to have to set a specific date, you know, to be able to say, yes, you meet the criteria. When you’re ready, give me a couple of weeks’ notice and we can reassess and I’ll do a final reassessment at that point. But, you know, yeah, why add that extra pressure onto people? (Medical Director & Practice Owner, GP)

### Experiences of AD at the ‘coal face’

This theme captured the variety of experiences of both AD providers and non-providers (encompassing a wide spectrum of views towards AD) when interacting with patients requesting AD.

#### Requests and communication

Some participants reported how they were used to patients asking for AD in the course of their practice, and that those conversations had now been made easier:

We’ve always had people request and ask, you know, that they would like us to end their life. So, it has changed those conversations because now there is, in some ways those conversations become a little easier because you can say, if this is something that you’re seriously considering here’s a pathway. Whereas before, you know, I do know I do remember things like patients who every day you would go in and they would ask, so have you got an injection for me today? You know, will you end my life today, over, even though they knew, and you’d say, you know I can’t do that. That’s not legal. (Specialist, Hospital (1))

One participant observed that patients’ understanding that they can end their lives is “the most amazing palliation” in itself (Specialist, Retired). Participants reported that patients who raised AD did not always pursue it further, if for example, their symptoms became manageable.

Some participants had different views on what constituted a request by a patient for AD. Similarly, there were divergent views regarding whether families who raise questions about AD should be referred to the co-ordinating body (SCENZ), or whether AD should only be discussed with patients. Another participant described how their workplace had created a card with information that could be handed over if a patient requested information about AD. However, participants reported a variation in how willing providers were to engage in discussion about AD with patients in general:

And so the party line here is for anyone who has a level of discomfort, is this is the SCENZ phone number, they will talk you through the process. Or raise it with your GP and if they can’t meet your need they will give you a SCENZ number and they will guide you through the process. And the conversation here, with families, is limited to non-existent depending on who the practitioner is that they disclose to. If they disclose to me, I am quite happy to just sit there and have a conversation about what I know and what they might experience. And what their expectations might be, and that kind of stuff. Others will just say, ring SCENZ. (Clinical Nurse Specialist, Hospice)

One participant also mentioned some instances of misinformation being communicated to patients:

… this patient was actively asking for assisted dying, [the clinician] said it takes a long time, it’s really difficult, you probably won’t ever manage to get it, it’s a really difficult process but what we can do is contact your GP, and tell your GP to organise a referral for you. So really wrong information … Nobody has to do a referral and they certainly don’t have to tell a patient who’s sick in hospital, who would never be able to get to a GP practice, to go and see their GP to get a referral. Or, ask a GP who’s not been involved in any of these discussions to try and send a referral. So, none of those things were appropriate. (Specialist, Hospital (1))

Several participants reported that one of the most difficult aspects of AD was telling people that they were ineligible. These participants described the distress of witnessing a patient’s suffering but not being in a position to relieve it:

I think one of the challenging things is finding people ineligible and communicating why that is. And trying to provide people with, you know, support. And, 'cause they’ve reached a point in their lives where they’re saying, you know, I’d like to have an assisted death, please … that’s quite a challenging conversation to have… people don’t come to this decision lightly. (Medical Director & Practice Owner, GP)

Because of the newness of AD, many participants reported how they were still working out “how to be” in a new clinical environment and learning by doing. This was particularly apparent when AD provider participants described their initial experiences of attending on the day to administer AD:

I found it so very difficult to decide on what I called end of life etiquette for the AMP. Just what do you say and how do you greet them and where and how do you behave and what do you do with all your, with your box of medications? And just how to make it all fit in with the event. And in particular, the greetings and how to interact with them, 'cause it is quite a different circumstance. It’s quite different. Yes, you’re there to help, but it’s in a way that none of us have helped people before. (Specialist, Hospital (2))

#### Pressure to ‘get it right’

A common theme for some AD provider participants was how keenly they felt a sense of responsibility for administering AD and the need to get it right on the day:

There’s still potential to just get it wrong and, you know, the last thing you want, the last thing you want to happen on that day is for things not to be what the family want. And there’s quite a lot of little potential pitfalls, I think”. (Specialist, Hospital (2))

The vast majority of patients undergoing AD choose to receive it via the intravenous (IV) route, which requires IV cannulation. Consequently, the practicalities of administering drugs were widely discussed by AD providers. One participant described how they practised cannulation prior to becoming an AD provider. Another described how in one case they were unable to get an IV line in, and the negative impact on themselves, the patient and their family. Yet another described the advice they provide to patients prior to AD to assist finding a vein: “You tell the person to drink a lot that morning and to keep warm” (GP, ARC).

#### Evolution of views

According to some participants, HCPs willingness to be involved in AD caring for a person undergoing AD may shift over time. Participants acknowledged that “you’re always going to learn from every single [AD] that you’re participating in” (Nursing Manager, ARC). This sentiment was echoed by those who were directly, peripherally, or not involved. AD was described as a “learning curve and a voyage of discovery” (Medical Director & Practice Owner, GP).

#### Stigma and secrecy

AD was often described as a largely taboo subject, even more so than ‘ordinary’ death:

We still don’t have a culture of discussing assisted dying freely, you know? Like, you can’t bring it up as a clinician, there seems to be like a bit of a taboo around it still. That’s my perception, you know, like yes, it’s there, but we pretend it doesn’t exist. (NP, Primary Care)

Some participants suggested that there is a need to normalise AD, a view influenced by the perceived negative consequences of stigmatising AD. For AD providers and supporters, some participants described a level of secrecy among peers. “I think people think it’s still a bit hush-hush, still a bit naughty. It’s a bit like abortion” (Specialist, Retired).

Some AD providers felt a strong need for privacy, especially those living and/or practising in small towns. For some, this was in part motivated by trying to avoid any difficulties that might arise for their immediate family members if they were associated with an assisted death in their local community. These AD providers found ways of managing this, such as only being involved in cases outside of their area (which often meant that they had to travel significant distances to patients) or only being involved in specific parts of the approval process).

Several participants described a patient’s decision to undertake AD being treated as “secret”, which caused both surprise and discomfort among staff. One participant, a hospital palliative care physician, described the following situation:

They’d been admitted for a couple of times on that ward for a number of weeks, and to have it suddenly confronted with, you have no idea this is happening on your shift today the staff said what was really challenging as well as one, not knowing so they couldn’t prepare themselves. They took them off the ward and then an empty bed came back, and that’s how they know that they’d had assisted dying. So, I think that’s a big challenge, understand that we want to keep this private and confidential so people can’t dissuade people or that if patients don’t feel comfortable of letting others know, but equally it has a big impact on the staff looking after them. (Specialist, Hospital (1))

For providers caring for patients, but not involved in assessing or providing AD, the perceived opacity of decision making and secrecy in some instances created moral distress, particularly if there was disagreement regarding eligibility decisions:

They have been under hospice care and I have questioned a) their prognosis and b) their cognition at times.

Interviewer: Were those patients found eligible for assisted dying services?

Yes which is why I’m concerned… [we identify] any staff who are troubled by experiences that they have. (Medical Director, Hospice (1))

While secrecy was a common theme, some participants described patients as being very open about receiving AD, which made it easier on HCPs. One participant noted the irony when a patient is embracing AD, while others may be distressed by the proposed AD:

The ones I’ve met so far are positively approaching this as a really positive last health care experience. They’re planning it, they’re implementing it. They’re making it their day. They’re making it their way. They’re being really clear about why they are accessing it and what the distress, what that is all about. (Clinical Nurse Specialist, Hospice)

### Functionality of the AD system

Participants commented on the functionality of various aspects of the AD system.

#### Access and timeliness

Some participants raised concerns about the timeliness of access to AD, and whether any delays occurred due to misinformation, dissuasion, or conscientious or institutional objection. Currently the MOH advises patients that the application process can take four to six weeks. While some participants thought this was a reasonable timeframe, others did not, especially when a patient’s death was imminent.

I do despair sometimes when clearly people, people who are clearly upset and, and in need of very rapid approach, get told in a blanket way, there’s a four-to-six-week process here. And that’s the last thing they want to hear when they’re a group who are being confronted by a person who’s on the verge of actively dying and they really want help. So, and so I think having a blanket message four to six weeks is not, is not a good approach and it needs to be tailored somehow, there needs to be an ability to say, to ask them what the speed of their needs are. (Specialist, Hospital (2))

Location was also identified as a factor in access, with the timeframe for rural residents compared to urban discussed:

One of the challenges is availability of practitioners and that, you know, sometimes we’ve got to travel down to the South Island because nobody else is available at the time they’re needed. And, for people with a tight prognosis time is of the essence. (NP, ARC)

Some participants noted the effect this may have on families as well as patients:

I think it just creates a lot of unnecessary stress for the families… about the logistics of the dying day in different places. And how horrible it can be for families, for even the person themselves. (GP)

#### Workforce capacity

A common issue raised by participants concerned barriers to accessing to AD, which was often attributed to the lack of AD providers. Many AD providers described delivering AD out of hours and in weekends, sometimes travelling significant distances to provide AD. In some cases, this required taking leave from their main employment, even forgoing their private clinics. Against this background participants discussed the challenge of how providers “look after themselves”, and how they “maintain their resilience” (NP, ARC).

Other factors considered to impact upon workforce capacity was the time required to undertake training (unpaid) and to deliver AD, as well as the wider clinical context (e.g., COVID-19, staffing shortages). Employment arrangements also affected whether clinicians could use work time to provide AD. Another potential reason for the lack of AD providers was the perception that some HCPs were ‘holding back’, waiting to see how well (or not) the system functions.

While some nurses provided information and supported patients considering or undergoing AD, both nurse and doctor participants thought that the nurse practitioner role should be expanded to use their skills to improve access to AD:

I think it was absolutely ludicrous that NP can only be brought at the end stage rather than at the assessment process. And, yet, we’re expecting them to do the prescription and then the administration. But, they haven’t been involved in the pre-lead to that, but then that is to do with their scope of practice and it will require a legislation change. (Nursing Manager, ARC)

#### Resources and training

Participants’ views on MoH resources and training were generally positive. Consistency of communications from the MOH and within organisations appeared to enable good training and implementation of resources.

Yeah so I mean there wasn’t much time, but, the Ministry of Health put together a really good set of resources. I’ve got to say, you know, they were really on to it. It’s not always the case. But, yeah they were. (Implementation Lead, Hospital)

Significantly, while some (non-AD provider) participants expressed concerns about the training of AD providers on specific aspects of the legislation, namely capacity assessment and prognosis, AD provider participants considered training on more practical issues such as putting in intravenous (IV) lines would have been beneficial (such in-person training was cancelled due to COVID-19).

Participants identified various aspects of the AD system in positive terms: the MOH’s principal clinical advisors (the first point of contact for patients and providers who contact the MoH) were described as being “helpful”, “efficient” and respectful of individual provider’s boundaries e.g. preferring not to provide AD services in their hometown. Accessing the MoH AD service to be assigned an AMP when patients need to be referred was reported as relatively easy and quick. The MOH-run peer support groups were well regarded, with participants appreciating the opportunity to learn from others’ experiences.

I think that the peer group and the support from the Ministry in coordinating that is going really well. The support from the Ministry of Health, the clinical nurse assistants I think they’re called there, works really well. They’re really good at their job. The process of getting referrals works well. You just get an email that says, you know, are you free to do this in this place? And, you know, you sort of accept or knock the referral so it’s, it’s not a high pressure. (GP & Practice Owner)

However, participants were critical of the IT system for managing the application process.

#### Funding

In an effort to enable equitable access, AD is publicly funded, including the cost of travel required for providers to deliver AD services in rural locations or urban areas where there is no available provider. AD providers are remunerated for each part of the process they complete, e.g. fees may be claimed for providing prognostic information; conducting a capacity assessment; or providing AD medication. In contrast, palliative care services do not receive specifically dedicated government funding, which was a point of contention for some participants:

But, it doesn’t seem to me very equitable that, that assisted dying gets major setup with pretty good funding… Yeah and if you look at the percentage of deaths under assisted dying it’s pretty well catered for. (Medical Director, Hospice (1))

Another participant offered a different perspective on the tension regarding funding of AD vis-a-via palliative care:

There’s been an inequity of funding [for end of life/palliative care] for a long time and that predates assisted dying …Okay you're giving all this money for this little service that a couple of hundred of New Zealanders will access per year. Whereas we’ve got all these other services, palliative care, end of life services which thousands of people need to access but they're poorly funded, so I could see where they [hospice and palliative care] get a bit ticked off with it… We need to argue that more funding is required, but it doesn't mean that it has to be at the cost of another service, you know. (Medical Director, Hospice (2))

## Discussion

This study is the first to explore the experiences of HCPs since AD became lawful in NZ. Overall, the findings indicate that the integration of AD services is still at a formative stage, with the diversity of opinions and experiences of participants interviewed suggesting that the difference in opinion regarding AD remains after the law came into effect. While some participants were generally supportive of AD and reported positive experiences with providing or facilitating it, others raised concerns about the practice of AD and the wider impact of legalisation. Although we did not initially seek participants’ views regarding the acceptability of AD, analysis of the data revealed that participants broadly fell into one of four groups: conscientious objectors; reluctant facilitators; AD allies; and lastly AD participators. While these categories may not be exhaustive, they provide a preliminary conceptual framework for examining health care providers’ experiences of, and engagement with, AD.

Consistent with a 2018 scoping review of HCPs experiences of AD implementation internationally, participants reported organisational, interprofessional, and individual challenges, during the early implementation of AD [17]. Like a recent qualitative study conducted in Victoria Australia, whilst practitioners providing AD reported positive experiences associated with what they generally considered constituted ‘patient-centred’ care, they also noted additional emotional, professional, and logistical demands of being an early AD provider [18]. Further, participants reported initial challenges interpreting and applying the law, a finding commonly reported in studies elsewhere [18, 19].

At the organisational level, a particularly striking finding of this study was the wide variation in organisational responses to AD that spanned from pro-active formulation and communication of AD policy and processes; to ambivalence regarding AD service involvement; to organisations claiming an IO to AD. While organisational non-participation is not uncommon in jurisdictions where AD is legalised, research indicates that it creates a ‘substantial burden’ for patients [19]. IO, as distinguished from mere ambivalence, describes an organisation refusing to provide what is otherwise a lawful service on grounds of conscience (i.e. the deeply held belief that AD is morally wrong). IO has emerged in many jurisdictions that have legalised AD. However, the concept has been subject to stringent criticism [20]. Public hospitals are essentially agents of the state, funded to provide lawful services; the notion that institutions possess a “conscience” is debated. However, the view that some organisations may legitimately claim a conscience-based objection regarding certain services has received support in New Zealand’s High Court [21]. In proceedings brought by Hospice NZ, an independent charitable organisation that receives partial public funding (approx. 50%), New Zealand’s High Court accepted that an organisation “may well have an entrenched ethos in which it operates", and that “so far as is practicable, an organisation should have the benefit of the right to freedom of conscience” [21]. This suggests that a hospice may exclude AD from the services it provides, and by extension may prevent HCPs it employs from providing AD services. However, it is difficult to justify similar conscience-based objections to AD in the case of institutions without such an organisational ethos or belief, for example non-denominational and publicly funded organisations [22]. While recent research illustrates the adverse impact of IO on patients and families [23] it is currently unclear whether some organisations in New Zealand are simply ambivalent as opposed to being unwilling to permit the provision of AD services in any circumstances on the grounds of ‘conscience’. Ambivalence is arguably an inadequate reason for failing to establish appropriate AD policy and processes [24].

Another important finding was the stigmatisation associated with requests for AD. While social stigma has been identified in the literature [25, 26], accounts of stigmatisation experienced by patients and families *within* the health care system itself are less common [27]. In this study some participants described how patients and families could feel shamed by HCPs for requesting AD and similarly, providers for offering AD. To avoid negativity or judgment, a patient’s choice to request and/or receive AD was sometimes not disclosed to other staff involved in the care of the patient, whether directly or indirectly. In some instances this secrecy caused distress for carers who only became aware of a patient’s plans to undertake AD after the fact. This dysfunctional process whereby stigma perpetuates shame and secrecy negatively impact patients and families. Ultimately stigma may reinforce secretive behaviour, with associated adverse effects on staff and impeding organisational learning [27]. While this may change with time as the service becomes more embedded, addressing this negative cycle requires a cultural shift in the way that AD is perceived and accommodated within health services.

Experiences elsewhere of implementing AD indicates that AD service provision often “matures” over time as health care providers’ attitudes evolve and systems and processes are established, embedded and improved [28–30]. Similarly in this study, participants reflected how their response to AD is evolving with experience. Incidental benefits of legalisation of AD were noted, such as the opportunity it provided to revisit and improve end of life policies and practice in regional hospitals, as well as being able to discuss end of life issues more openly. Similar to other studies, a strong theme with both providers and non-providers was the importance of patients receiving good end of life care [31]. For some participants in this study, AD ideally requires personnel skilled in palliative care because they are better skilled at prognosticating, undertaking capacity assessments, and providing bereavement care. However there is a tension when, as is reflected in the literature, palliative care providers as a group are less supportive of AD than other health professionals [32]. In general, New Zealand AD providers were positive regarding the resources and training provided by the MOH [9]. However, similar to other studies, AD providers expressed concern regarding the capacity of the currently small AD workforce to provide equitable services throughout NZ [18].

### Limitations

A strength of this study is that it includes HCPs with a diversity of perspectives on AD. This, too, is a limitation in that the views presented by participants were often described through the lens of their personal position on AD. Also, some evaluation of particular cases may also be incomplete, for example, where a participant was not involved in assessing a patient for AD, they may be relying on incomplete or second-hand information. Another limitation of the study is that it involved mainstream health organisations and does not specifically reflect Māori cultural viewpoints on AD.

This study involved both individual interviews and a focus group. It is possible that the focus group discussion may have been constrained due to the workplace setting and social norms. However, the opportunity to provide further comments via email sought to ameliorate any potential power imbalance or other factors that might have inhibited participants. Significantly, there were limitations on the diversity within our participant group as most participants were New Zealand European or European, with only 3 Chinese, 1 Māori, 1 Samoan, and 1 ‘Other’ participant. We did not examine the role of participants’ religion or culture in their views as it was beyond the scope of the study. A further limitation is that this study captures the point-in-time experiences of HCP in the very early stages after AD became lawful in New Zealand and AD systems evolve over time. Finally, this study does not include the perspectives of families and patients, although further research is planned that will involve a wider group of stakeholders.

## Conclusion

Overall, this study affirms that AD represents a major shift in end-of-life care, requiring ongoing education of the health care work force and transparency regarding AD policies and processes. Like other studies, this research indicates that implementing a new law permitting AD is an ongoing and evolving process; currently there is a wide variation in organisational approaches to AD, with many organisations still establishing and refining AD policies. In particular, this study suggests that underlying attitudes to AD may impact the experience of, and engagement with, the implementation of AD. A significant finding was the reports of AD stigmatisation within the health system, which was associated with keeping the AD process “secret”, with adverse effects on HCPs involved in caring for that patient. Further research into the factors that influence HCPs responses to, and experience of, the integration of AD into clinical practice, as well as the extent to which HCP may move between categories over time, would be beneficial. In addition, further research into the impact of IO as well as the integration of AD within Māori health organisations is required.

## Supplementary Information

**Additional file 1.**

## Data Availability

The datasets generated during and/or analysed during the current study are not publicly available due to participant confidentiality but are available from the corresponding author on reasonable request.

## Abbreviations

AD: Assisted dying
AMP: Attending medical practitioner
ARC: Aged residential care
NZ: Aotearoa New Zealand
GP: General practitioner
HCPs: Health care providers
IMP: Independent medical practitioner
IV: Intravenous
IO: Institutional objection
MoH: Ministry of Health
NP: Nurse practitioner
SCENZ: Support and Consultation for End of Life in New Zealand
the Act: End of Life Choice Act 2019
